# Spatial Distribution and Temporal Variability of Ammonium-Nitrogen, Phosphorus, and Potassium in a Rice Field in Corrientes, Argentina

**DOI:** 10.1155/2014/135906

**Published:** 2014-12-31

**Authors:** Luis Alberto Morales, Eva Vidal Vázquez, Jorge Paz-Ferreiro

**Affiliations:** ^1^Faculty of Agricultural Sciences, National University of the Northeast, Sargento Cabral 2131, 3400 Corrientes, Argentina; ^2^Faculty of Sciences, University of A Coruña, Zapateira Campus, 15008 A Coruña, Spain

## Abstract

Proper and effective management of soil nutrients requires assessment of their variability at the field scale. We compare the effects of lime amendment rate on the spatial variability of three macronutrient forms (NH_4_^+^-N, Olsen P, and Mehlich-1 K) in a paddy soil at three different dates during the growth period of a rice crop. The field work was carried out near Corrientes, Argentina. Lime treatments were 0, 625, and 1250 kg ha^−1^ dolomite, and each liming dose was applied to a 1.7 ha field. Ninety-three soil samples per treatment were first collected in aerobic conditions and then two more times after flooding, at bunch formation and flowering. Soil NH_4_^+^-N increased along time, whereas P was highest at bunch formation and K steadily decreased along the rice growth period. Dolomite addition increased macronutrient availability at the first and second samplings, but its effects at the third sampling depended on the element. The three soil nutrients analyzed displayed strong patterns of spatial dependence for the three lime treatments and at the three periods studied. The areas with relative high or low macronutrient concentrations within each field were not stable throughout the rice growth period. Seasonality in the spatial distribution of macronutrients may be of agronomic value for site specific management.

## 1. Introduction

Macronutrients (N, P and K) play an important role in crop production. Inadequate fertilizer management limits crop yield, results in nutrient mining, and causes loss of soil productivity. A satisfactory nutrient status saves the soil, a limited natural resource, and also prevents environmental pollution [1]. Proper and effective management of macronutrients and assessment of their effects on environmental quality requires an understanding of their variability in concentration across the fields. Moreover, knowledge about spatial and temporal behaviour in the variability of nutrient status is the key for site specific management trough precision agriculture techniques. Site specific management can help to minimize fertilizer inputs thought the whole field without prejudicing yields, which would contribute to bridge economic with environmental advantages [2, 3].

The spatial variability of soil properties is the outcome of the interaction of several soil forming factors and processes and in agricultural and forest fields involves also effects of management practices [3–5]. There has been a growing interest in the study of the spatial variability of soil characteristics, including macronutrients, using geostatistics since the 1980s [4–8]. More recently, the value of spatial measurements of soil properties coupled with geostatistical techniques to develop site specific management practices also has been widely acknowledged [2, 9, 10]. Indeed, geostatistical analysis has been carried out to assess heterogeneity in paddy soils, focussing either on soil properties and/or crop yield [5, 11] or on soil properties and/or nutrients [12–17].

In paddy soils, spatial variability has been shown to occur over distances of meters or tens of meters. Because these soils are characterized by a relatively flat topography and seasonal flooding, in the past they have been thought to be apparently homogeneous. However, large spatial variability in soil properties has been reported for paddy fields of different countries [5, 11, 12, 14, 16], even if at a first sight this might be considered as a quite unexpected result. However, more recently it has also been shown that factors such as small differences in elevation after land-levelling [18] and redistribution of soil components and/or nutrients by lateral water movement across neighbouring small fields surrounded by ridges [19, 20] may drive heterogeneity of crop yield and soil properties at different scales. Temporal oscillations in the spatial patterns of variability of soil properties or soil nutrients in rice fields have been also previously reported [12, 13, 16, 20], even if this issue has been less frequently addressed.

Successful rice production requires an adequate soil fertility status and especially satisfactory levels of N, P, and K. In flooded soils major chemical and electrochemical changes occur that have a profound influence on soil nutrient availability. In this way, under submerged condition as in paddy rice cultivation ammonium (NH_4_^+^), rather than nitrate (NO_3_^−^), has been found to be the main source of N for crop growth. This is because NO_3_^−^ (if present) can be depleted rapidly after flooding due to denitrification, while NH_4_^+^ tends to accumulate with time of submergence due to lack of O_2_ for nitrification. Therefore, ammonium is likely to be the main form of nitrogen in flooded soils [21, 22]. Both forms of nitrogen, but especially NO_3_^−^, are very mobile and may show a marked seasonal change in concentrations during the growing season, owing to the unstable physicochemical properties, for example, redox potential, or to variations in microbial activity.

Phosphorus availability to rice mainly depends on soil pH. Flooding increases pH of acid soils and decreases pH of calcareous and sodic soils. In acid soils P is associated with Fe and Al compounds, whereas in soils, with pH higher than 6.5, P is primarily associated with calcium and magnesium. Flooding rice soils generally moderates the pH towards a neutral pH condition and consequently the availability of P is promoted [23, 24].

Several forms of soil potassium have been identified in paddy soils. Exchangeable K is considered mostly readily available, but other slowly available forms, which mainly depend on clay mineralogy and physicochemical properties, have been also identified in paddy soils [25]. The removal rate of K with rice grain and straw is rather high. Notwithstanding, either paddy farmers do not use K fertilizers or the used amount is insufficient to balance K removal [26]. Therefore, K availability of flooded rice soils is mainly affected by fertilizer application and straw management practices. Therefore, K deficiencies have been found to occur, even if only to a limited extent, in several types of lowland rice soils in association with small concentrations of exchangeable K, or to inactivation of K release by clay components.

Soil variability occurs across a continuum of spatial and temporal scales. Nutrient maps based on intensive soil sampling are a means to implement field-proven decision criteria for managing precision fertilizer input [5, 27, 28]. Experimental work was conducted on a paddy field in Corrientes, Argentina, to test the effect of lime amendment on the spatial distribution patterns of soil pH and Eh and on macro- and micronutrients. Three successive sampling campaigns were carried out to assess the stability of the spatial distribution along the growing season of the studied soil properties. Results reported on previous work for pH and Eh [17] and for extractable Fe, Mn, and Zn [12] showed a rather strong seasonality in the spatial distribution of these variables with changing patterns between sampling dates. Seasonal stability of nutrient maps has been considered as a prerequisite for efficient precision agriculture management [20]. On the other hand, several soil properties and nutrient in paddy soils previously have been shown to vary along the rice growing period [12, 17, 22, 24, 25]. Therefore, the first objective of this study was to conduct a field-scale analysis of the spatial variability of ammonium-N and available phosphorus and potassium, using the unpublished data sets of the above mentioned trial. The second objective was to investigate stability in the spatial distributions of ammonium-N, Olsen P, and Mehlich-1 K along the rice growth season.

## 2. Material and Methods

### 2.1. Site, Sampling Collection, and Laboratory Analysis

The study was conducted on a paddy field located near Corrientes, Argentina, cropped to rice (*Oryza sativa*, L.). The experimental site and the sampling procedure have been described before [12, 17] and therefore this information will be briefly summarized here. The climate is warm, subtropical with mean temperature of 20.1°C and mean yearly rainfall of about 1200 mm. The soil was classified as a typic Plintacualf [29], and it was an acidic soil (pH = 3.7, before liming), silt-loamy textured.

**Figure 1:**
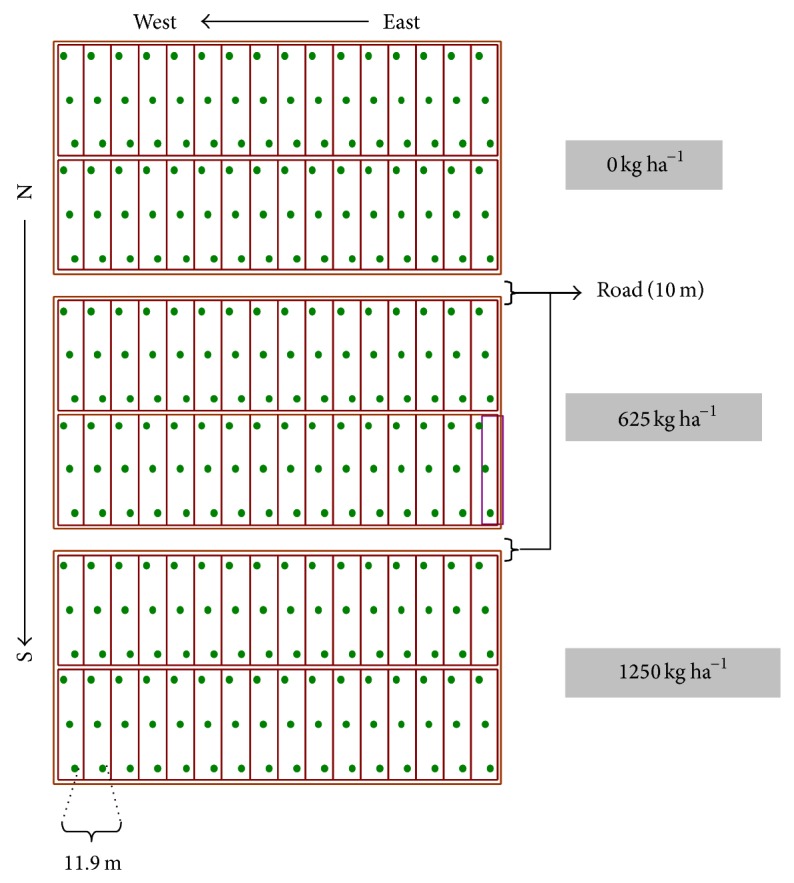
Sampling grid with 96 sampling positions per treatment.

A field experiment was conducted to assess the influence of liming on several soil properties. The study site was an irrigated field with two previous years under lowland rice when this study was started. The entire field was subdivided in three parcels of 1.7 ha surface each, which received dolomite amendments in amounts of 0 (control), 625 kg ha^−1^, and 1250 kg ha^−1^. Each parcel was surrounded by a 10 m wide road and enclosed 32 plots of 50 m × 11.9 m, limited by 1.9 m width water channels (Figure 1). The experimental field was fertilized at sowing time using an N-P-K mixture with 35 kg of urea, 47 kg of superphosphate, and 95 kg of KCl. The fertilizer was uniformly broadcasted over the rice field.

Soil samples were collected at three different stages during the rice growth period. The first sampling was performed in aerobic conditions, just before sowing, the second sampling at bunch formation, that is, 28 days after flooding, and the third sampling at flowering stage, that is, 56 days after flooding. Ninety-three samples per liming treatment were taken at each of the three sampling dates. The basic sample grid was 11.9 × 20 m (Figure 1). Therefore, three soil samples were taken from each of the 32 single plots of a given parcel or treatment.

The soil was collected to a depth of 15 cm. Soil samples were air-dried and sieved (2 mm mesh). Ammonium-N was extracted with 2 M KCl and determined using methods described in [30]. Available P was extracted using bicarbonate [31]; phosphorus concentrations were determined colorimetrically using ascorbic acid-ammonium molybdate reagents. Available K was extracted with the Mehlich-1 solution [32] and measured by atomic absorption spectrometry.

Mean values of pH and redox potential (Eh), reported in [17], are summarized in Table 1. On average, mean pH increased by 2.3 units along the rice growth period. Moreover, as expected, mean pH ranked as control <625 kg ha^−1^ dolomite <1250 kg ha^−1^ dolomite, at the three sampling dates. Redox potential decreased along the rice growth period with increasing duration of the anaerobic conditions; the effect of dolomite addition was to decrease mean soil redox potential on the three sampling dates.

### 2.2. Statistical and Geostatistical Analysis

The 27 data sets studied (3 nutrients × 3 lime treatments × 3 sampling dates) were first analyzed for normality by means of the Kolmogorov-Smirnov test. Then, descriptive statistics including mean, variance, coefficient of variation, maximum, minimum, skewness, and kurtosis were determined. Pearson correlation coefficients were also calculated to determine the relationship between soil properties.

Geostatistical analysis is based on the assumption that measurements separated by small distances are more likely to be similar to each other than those farther apart, meaning that spatial autocorrelation exists. This hypothesis can be verified through examination of semivariograms for the attributes under investigation. Moreover, the statistical tool used to measure the autocorrelation between samples is called the semivariogram. An experimental semivariogram can be obtained from semivariance values calculated as a function of the distance, *γ*(*h*), which are given by the following equation:

(1) $$\begin{array}{c} \gamma \left(h\right)=\frac{1}{2N\left(h\right)}\overset{N(h)}{\underset{i=1}{\sum }}{\left[Z\left(x_{i}\right)-Z\left(x_{i}+h\right)\right]}^{2}, \end{array}$$

where *Z*(*x*_*i*_) and *Z*(*x*_*i*_ + *h*) are the actual values of the variable, *Z*, at places *x*_*i*_ and (*x*_*i*_ + *h*), and *N*(*h*) is the total number of data pairs separated by a distance *h*. Thus, the experimental or sample variogram is obtained by changing *h*.

The experimental semivariogram must be fitted by a model that mathematically describes the spatial variation. Several standard models (i.e., spherical, exponential, Gaussian, etc.) are currently available. In this work, calculation of sample variogram and fitting of models were made using accredited criteria and procedures [2, 33]. The cross-validation technique was used for model fitting [34]. Then, agreement between experimental and modelled semivariograms was judged by various indicators, including determination coefficient (*r* ^2^), mean error (ME), and no dimensional mean square error (NMSE).

Semivariogram models were described using the following basic parameters: the distance *h*, the sill variance (*C*_0_ + *C*_1_), and the range of spatial dependence (*a*). The sill variance eventually reaches an upper bound; this parameter consists of a spatially correlated or structural variance (*C*_1_) and may include a nugget variance (*C*_0_), which represents the variation that has not been resolved at the scale of sampling. The degree of spatial dependence can be expressed by the ratio of nugget variance (*C*_0_) to the threshold (*C*_0_ + *C*_1_) variance. The nugget to sill ratio was used to qualitatively classify the spatial dependence into strong (<25%), moderate (25 to 75%), and weak (>75%) following accredited criteria [6].

Based on the best fitting semivariogram model, the kriging approximation was used for interpolation and mapping purposes. Kriging is an optimal method of prediction in the sense that it is unbiased and the predictions have minimum variance [27, 28].

## 3. Results and Discussion

### 3.1. Statistical Analysis of Soil Macronutrients

Descriptive summary statistics of the 27 data sets studied are presented in Table 2. Concentrations of soil NH_4_^+^-N just before flooding ranged from 20.07 to 28.87 mg kg^−1^ and those at rice flowering (56 days after flooding) ranged from 37.74 to 43.92 mg kg^−1^. The latter were significantly higher than the former (*P* < 0.05) and this was for the three liming treatments. The average increase in NH_4_^+^-N concentrations between the first and the third sampling period was 88%. Ammonium-N accumulation in anaerobic conditions is an expected result, due to the lack of conversion of organic N into mineral N in paddy soils [21, 22], as stated before. However the amounts of ammonium and nitrate nitrogen determined at the second sampling date, which ranged from 24.73 to 28.50 mg kg^−1^, were not much different from those of the first sampling date, even if the rate of NH_4_^+^-N accumulation was not negligible for the control treatment during this period. Moreover, comparison between the first and the second sampling dates showed small but significant (*P* < 0.05) ammonium-N losses and increases at the 625 kg ha^−1^ and 1250 kg ha^−1^ treatments, respectively. The net increase in NH_4_^+^-N after flooding is the result of the net balance from accumulation (owing to the lack of O_2_ for nitrification) and losses (owing mainly to plant uptake but also to denitrification processes).

There were positive effects of lime amendment on NH_4_^+^-N concentration, both at sowing and at bunch formation, as the differences between the control treatment and the two treatments amended with dolomite were significant (*P* < 0.05). Nevertheless, concentrations of NH_4_^+^-N within each sampling period were considerably stable, as shown by the smaller range of values among liming treatments than among sampling dates.

Coefficients of variation of the NH_4_^+^-N data sets studied ranged from 9.7 to 20.3% and showed a trend to decrease for the successive sampling periods. This seasonal reduction in the statistical variability of NH_4_^+^-N concentrations throughout the rice growth period could be the result of more uniformity in microbiological activity across the field, which in turn may be due to a higher homogeneity of the redox conditions (Table 1). All the ammonium-N data sets studied were normally distributed. These distributions were slightly negatively or positively skewed, as the skewness parameter ranged from −0.462 to 0.654.

**Figure 2:**
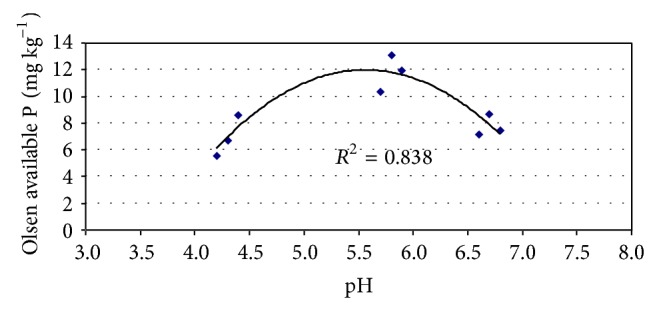
Relationship between average pH and average Olsen extractable phosphorus.

The mean concentrations of Olsen extractable P ranged from 5.52 to 8.57 mg kg^−1^ at the first sampling, in aerobiosis, from 10.36 to 13.12 mg kg^−1^ at the second sampling, 28 days after flooding, and from 7.14 to 8.67 mg kg^−1^ at the third sampling, 56 days after flooding. These results are in accordance with the fact that P availability has been found to be optimal when the soil pH is between 6.0 and 6.5.  Figure 2 shows the relationships between mean pH and mean Olsen available P in the studied treatments. The initial concentrations of Olsen P in the very acid soil (in anaerobic conditions) were low [24, 31], but these increased rapidly after flooding, in all the liming treatments. Thus Olsen P increased with increasing soil pH and was highest for mean pH values between 5.7 and 5.9 recorded at the second sampling period. Subsequently, concentrations of Olsen P measured in the third period were lower compared to the second period, in accordance with the mean pH values ranging from 6.6 to 6.8. Therefore, the highest available P concentrations were recorded at bunch formation, owing to soil pH ranges near optimum values [24, 31]. However, available P was depleted throughout the late growing season, owing mainly to pH increases above to the 6.5 threshold, but also to the plant uptake.

Liming significantly (*P* < 0.05) increased Olsen P availability in aerobic conditions before flooding. Also, at the second sampling the mean concentration of Olsen P was significantly lower (*P* < 0.05) in the control treatment than in the two dolomite amended treatments. However, at the third sampling date, the highest mean Olsen P values were recorded at the 625 kg ha^−1^ dolomite treatment. In general, and in agreement with the trends observed before for ammonium-N, Olsen P concentrations showed smaller differences among liming treatments than among sampling dates (Figure 2).

Coefficients of variation for Olsen P ranged from 15.4 to 25.8% and, therefore, showed a similar order of magnitude for the different treatments and sampling dates studied. The seasonal stability in the statistical variability of available P probably reflects the influence of pH as main factor driving the dynamics of P in paddy rice soils. All the Olsen P data sets studied also were normally distributed. The coefficients of skewness for these distributions ranged from 0.169 to 1.069 and were positive for 8 out of 9 data sets. Positively skewed distributions indicate the presence of some few extreme high values of available P.

The critical value of Olsen extractable P across a wide range of soil types has been established at 6.0 mg kg^−1^ [23]. Subcritical P levels are expected to cause reductions of production under intensive rice cropping. Available phosphorus concentrations before flooding ranged from very low to low and become moderate after the additional release promoted by the soil pH increase due to flooding. No visual symptoms of P deficiencies were observed in the rice crop studied. Therefore, the applied supply of P, as superphosphate, may be sufficient for the agricultural practices and rice production levels of the local area. Moreover, the 625 kg ha^−1^ dolomite treatment was more efficient than the 1250 kg ha^−1^ dolomite treatment for releasing available Olsen P, in anaerobic conditions, as shown by mean values at the second and third sampling dates.

Concentrations of available K extracted with Mehlich-1 are reported in Table 2 and ranged from 28.15 to 50.41 mg kg^−1^, 32.09 to 40.44 mg kg^−1^, and 17.25 to 19.41 mg kg^−1^ at the first, second, and third sampling dates, respectively. Therefore, available K concentrations steadily decreased throughout the crop period, from sowing to bunch formation and then to flowering. On average Mehlich-1 extractable K was more than two times higher just before flooding than after 56 days of submersion. Available K depletion probably was mainly due to the plant uptake, so that it is expected to increase again after rice harvesting and straw mineralization. Notwithstanding, the dynamic of K along the rice vegetative period could be also affected by soil intrinsic factors such as clay mineralogical composition and physicochemical characteristics.

Mean extractable K concentrations in the soil measured at sowing and at bunch formation also increased significantly (*P* < 0.05) with rate of dolomite application. Hence, in the two first sampling dates, mean Mehlich-1 K values for the three dolomite treatments ranked as control < 625 kg ha^−1^ < 1250 kg ha^−1^. At the third sampling date, however, the mean extractable K concentrations measured at the control treatment were significantly lower than those at the two dolomite amended treatments, but no significant differences were found between the two treatments with dolomite amendment. The control treatment had the lowest mean extractable K level before flooding and the highest after 58 days of flooding; in contrast the 1250 kg ha^−1^ treatment had the highest mean extractable K level before flooding and the lowest after 58 days of flooding. Hence, the rate of diminution in soil available K throughout the crop growth period was greater for the dolomite amended treatments than for the control treatment. Therefore, seasonal changes in available K under paddy rice cultivation were not similar for all the treatments, suggesting specific K fixation or depletion depending on soil reaction.

Coefficients of variation for Mehlich-1 K have been found to vary between 11.4 and 25.6%, and they were smaller than 20% in eight out of nine data sets. On average, CVs for Mehlich-1 extractable K were lower than those obtained for Olsen P. Again, all the available K data sets studied were normally distributed. Moreover, the distributions of extractable K for all the data sets analyzed were positively skewed, with skewness coefficients ranging from 0.004 to 1.012. The highest values of extractable K exhibited by distributions with the largest skewness coefficients may be associated with locations with the highest straw residues left after crop harvesting.

Mehlich-1 extractable K concentrations below 50 mg kg^−1^ are considered to be low for an intensive high-yielding rice crop [28]. Thus, according to these soil tests, the amount of K fertilizer applied was not sufficient to maintain a good nutritional status during the entire growth season. No visual deficiencies were detected for K in our experimental field. Consequently, the applied K can be considered to be sufficient to maintain current rice yields of about 4–6 t ha^−1^ in the studied area.

### 3.2. Spatial Dependence and Kriging Maps of Soil Macronutrients

**Figure 3:**
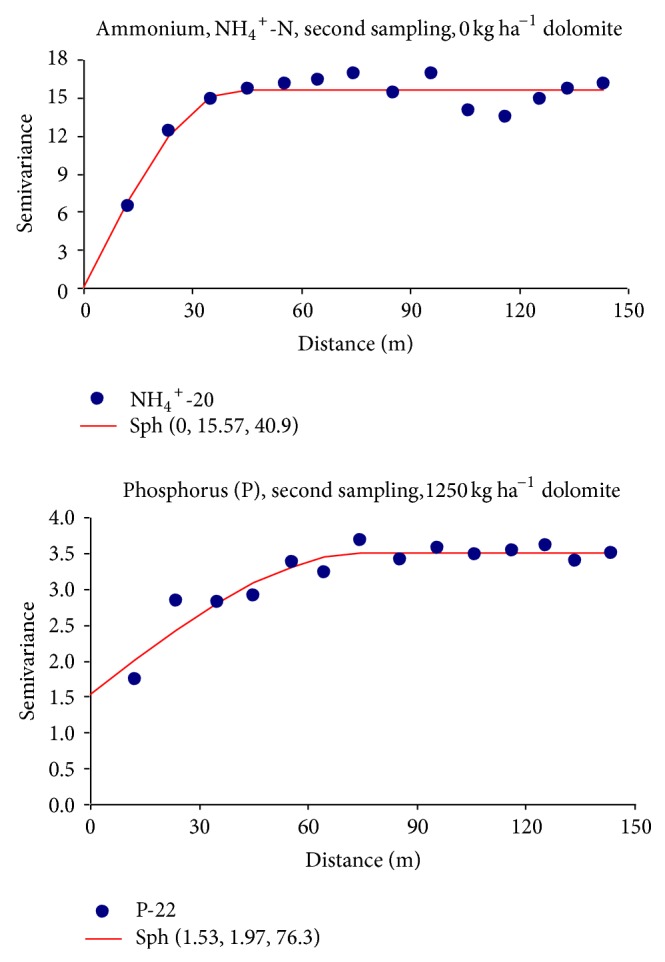
Selected examples of semivariograms corresponding to experimental data sets without nugget effect and with the highest nugget effect modelled.

Selected examples of semivariograms are presented in Figure 3. They correspond to one of the data sets with the highest degree of spatial dependence (no nugget effect) and to the data set with the lowest degrees of spatial dependence, meaning the highest nugget to sill ratio, that is, (*C*_0_)/(*C*_0_ + *C*_1_). The geostatistical parameters for the best fitted semivariogram models describing the spatial dependence of the soil macronutrients studied are listed in Table 3, together with the cross-validation parameters used for assessing the goodness of fit. All the semivariograms modelled increased with lag distance (*h*), until a more or less constant value was reached, at a given separation distance, that is, the sill or total semivariance (*C* + *C*_0_); this separation distance (*a*) is called the range of spatial dependence. Note that the nugget variance, *C*_0_, is the semivariance at *h* = 0. Experimental semivariograms of the NH_4_^+^-N concentrations measured at the three lime treatments and during the three periods studied could be described quite well by either spherical or exponential models with a nugget component (*C*_0_) plus a spatial component (*C*_1_) with a range of spatial dependence varying between 40.9 and 84.6 m. For Olsen P and Mehlich-1 K, all experimental semivariograms were described by spherical models with a range between 51.4 and 76.8 m.

Theoretical models were fitted to experimental semivariograms based both on subjective visual inspection and on the following parameters obtained by cross-validation: determination coefficients of the regression between calculated and kriging estimated values (*r* ^2^), mean errors (ME), and no dimensional mean square errors (NMSE). Goodness of fit was assessed by proximity to ideal values of these parameters for a perfect fit, that is; *r* ^2^ = 1, ME = 0, and NMSE = 1 [12, 33]. Determination coefficients were *r* ^2^ ≥ 0.639, mean errors were ME ≤ 0.059, and no dimensional mean square errors were 0.813 < NMSE < 1.189. Therefore, the values of these parameters suggest that all models adequately fitted the spatial dependence of the studied macronutrient data sets.

Note also that in most of the cases studied the experimental semivariograms were best described by a spherical model. The exception was for two NH_4_^+^-N data sets corresponding to the 625 kg ha^−1^ and 1250 kg ha^−1^ treatments during the second sampling, which were best fitted by exponential models. Other soil properties studied before in our experimental field (pH, Eh, extractable Fe, Mn, and Zn) also have been found to fit mostly spherical models and otherwise exponential models [12, 13, 17].

The nugget variance represents either experimental variability induced by potential laboratory errors or field random variability that can not be detected at the scale of sampling. The nugget to sill ratio, *C*_0_/(*C* + *C*_0_), ranged between 0 and 43.7% for all the data sets studied. This ratio was zero at four data sets, was smaller than 25% at 11 data sets, and ranged from 25 to 50% at 12 data sets, indicating in most of the cases a strong to moderate degree of spatial dependence, according to the authorized criteria [6] previously quoted. Moreover, the magnitude of the nugget variance showed no dependence of the liming treatment or the sampling date. Altogether, the small to moderate nugget effects indicate that the sampling grid used was proper to reflect the spatial dependence of the studied macronutrients.

The sill value (*C* + *C*_0_) reflects the total variance for very large distances; the sill is given by the sum of the nugget semivariance (*C*), which may be zero, plus the portion of the semivariance that is spatially structured (*C*_0_). As expected, for each data set, the value of the sill modeled was of the same order of magnitude as the value of statistical variance. The sill value ranged from 12.35 to 32.63 (mg kg^−1^)^2^ for NH_4_^+^-N, from 1.82 to 6.67 (mg kg^−1^)^2^ for Olsen P, and from 6.17 to 89.81 (mg kg^−1^)^2^ for Mehlich-1 K, indicating that Olsen P had the weakest pattern of spatial variability. Mean sill values were lowest for NH_4_^+^-N at the second sampling date; however in this sampling they were highest for available P and K. Main factors responsible for the differences in semivariance for very large distances, that is, for the sill value, of the data sets studied here could be: (a) seasonal variations, driven by the different biogeochemical processes during aerobic and anaerobic conditions, (b) nutrient uptake by the rice crop, and (c) management effects such as liming.

The values for the range of spatial dependence of the semivariogram models were of the same order of magnitude for the three nutrients studied, varying from 40.9 to 84.6 m for NH_4_^+^-N, from 47.7 to 76.3 m for Olsen P, and from 51.4 to 76.8 m for Mehlich-1 K. Highest values of this parameter were about two times larger than smallest values. The range of spatial dependence showed a slight trend to increase throughout the rice growth period. This suggests the area of similarity of the nutrient content becomes larger during the rice growing season. Note also that this area may embrace several individual rice plots of 50 × 10 m size (Figure 1). Moreover, the values of the range of spatial dependence for ammonium-N and extractable P and K were also similar to those previously reported for pH and Eh [17] and for extractable Fe, Mn, and Zn [12, 13] at the same experimental field. Sample locations separated by distances smaller than the range are more alike and are spatially correlated, whereas those separated by distances greater than the range are spatially uncorrelated. Summarizing, there were some similarities in the range of spatial dependence for soil chemical properties measured under different lime treatments at different sampling dates, but differences in range also were found with the same frequency as similarities.

Spatial dependence at paddy fields of various sizes [5, 11–15] and also at the scale of a whole district cropped with rice [16] has been reported for several soil properties. Semivariograms depend on the sampling scale, sampling design, and support of the underlying data sets; thus, there is no “absolute” semivariogram for a soil property. Since all these factors vary between published studies, it becomes difficult to compare the results by different authors. Moreover, in our experimental field the soil properties analysed in the present and in previous work, namely, pH, Eh, and macro- and micronutrient concentrations [12, 13, 17], showed seasonal modifications in the patterns of spatial dependence. A seasonal pattern of variability, which may depend upon local conditions, is an additional drawback to compare results from different filed trials.

Until now, the temporal oscillation in the variability of soil properties has mainly been reported for soil biological properties, as, for example, soil enzymes, soil biological activity, and soil fauna, and for soil quality indices based on these properties [35, 36]. However, in this work and in previous work carried out at our experimental field [12, 13, 17], we described seasonality in the spatial patterns of variability of soil general properties or soil nutrient content in rice fields, which has been also demonstrated by field trials conducted in other different areas [20, 37].

Spatial variability of soil properties depends both on soil forming factors and processes and on management practices, as stated before. In our case study, climate, topography, and water level were homogeneous throughout the experimental fields. The parent material consisted of sedimentary rocks characterised by various particle size distributions, which could draw spatial variability at the decameter scale [12]. Element speciation and concentrations of macro- and micronutrients at a given location within a field also depend on various soil properties, such as organic matter content, total element composition, pH, and redox potential (Eh), on plant nutrient uptake, and on management practices. In a previous work pH and Eh of the studied field have been demonstrated to undergo seasonal variability. On the other hand, the fertilizer use efficiency of rice is very low when all fertilizers are applied as base dressing, as in this field experiment; therefore nutrient uptake by rice plants cannot be viewed as a major source of variability in our conditions. However, current management practices of the rice crop, associated with the irrigation system employed, may result in uneven water application [20] and also may drive redistribution of elements by lateral flow across the smallest production units [19, 20]. Therefore redistribution by unsteady flooding and lateral water movement could be considered as a main source of spatial variability, even if all the experimental units were managed similarly. In addition the role of microrelief heterogeneities has been also stressed as an essential source of variability in soil properties under rice cultivation [20, 37].

Therefore, the results of this work together with those reported in previous work [12, 13, 17] suggest that texture, soil mineral and organic composition, and liming dose may be possible factors influencing the spatial variability of soil macronutrients at the studied parcel. In addition, seasonality or temporal variability of the patterns of spatial variability of the studied macronutrients and other soil properties may be mainly driven by uneven flooding associated with irrigation, lateral flow between small plots, and microtopographic irregularities.

**Figure 4:**
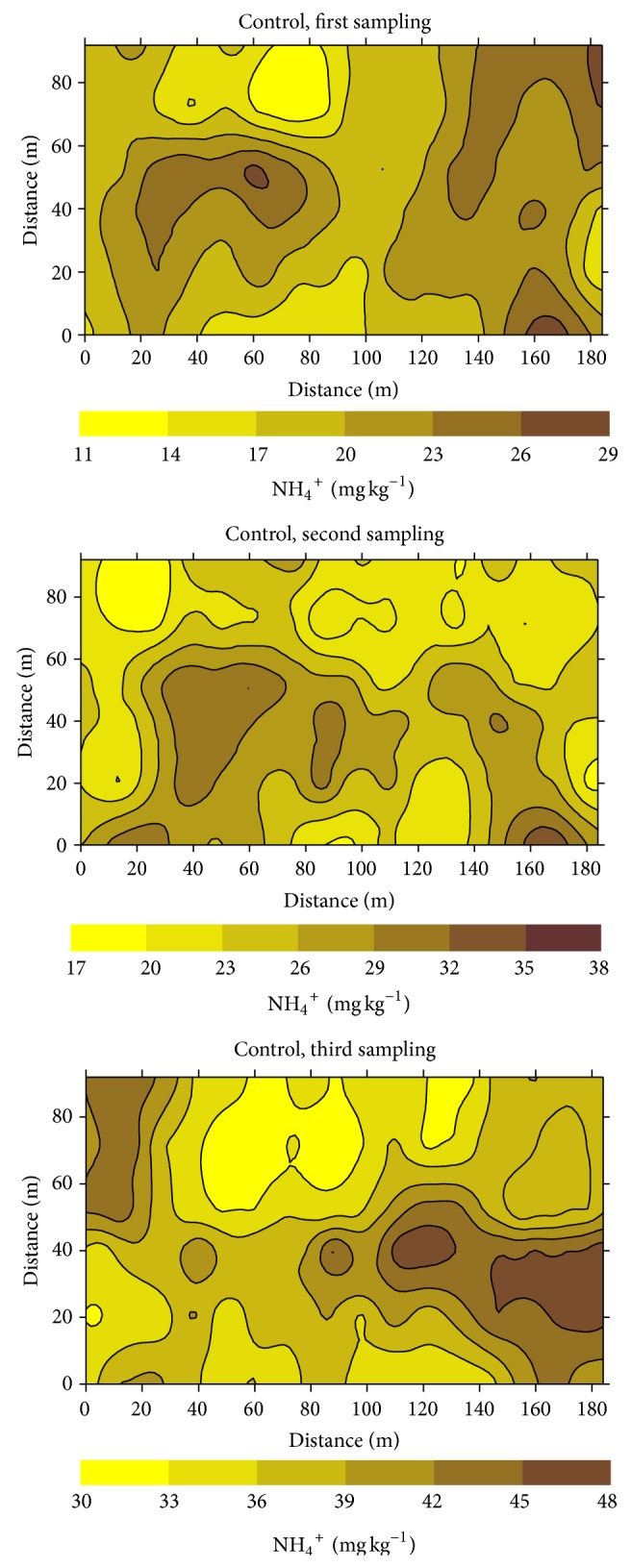
Kriging maps for ammonium-nitrogen (NH_4_^+^-N) at the control treatment, for successive sampling dates.

**Figure 5:**
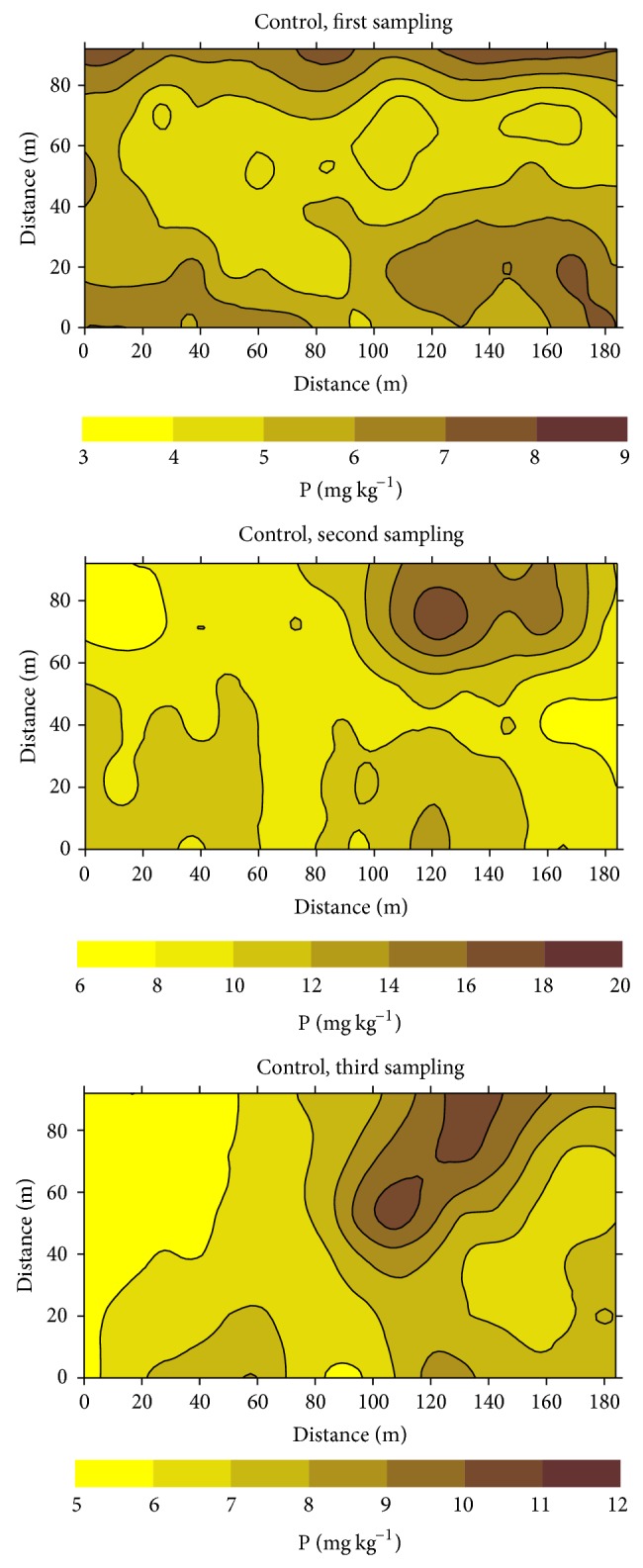
Kriging maps for Olsen extractable phosphorus (P) at the control treatment, for successive sampling dates.

**Figure 6:**
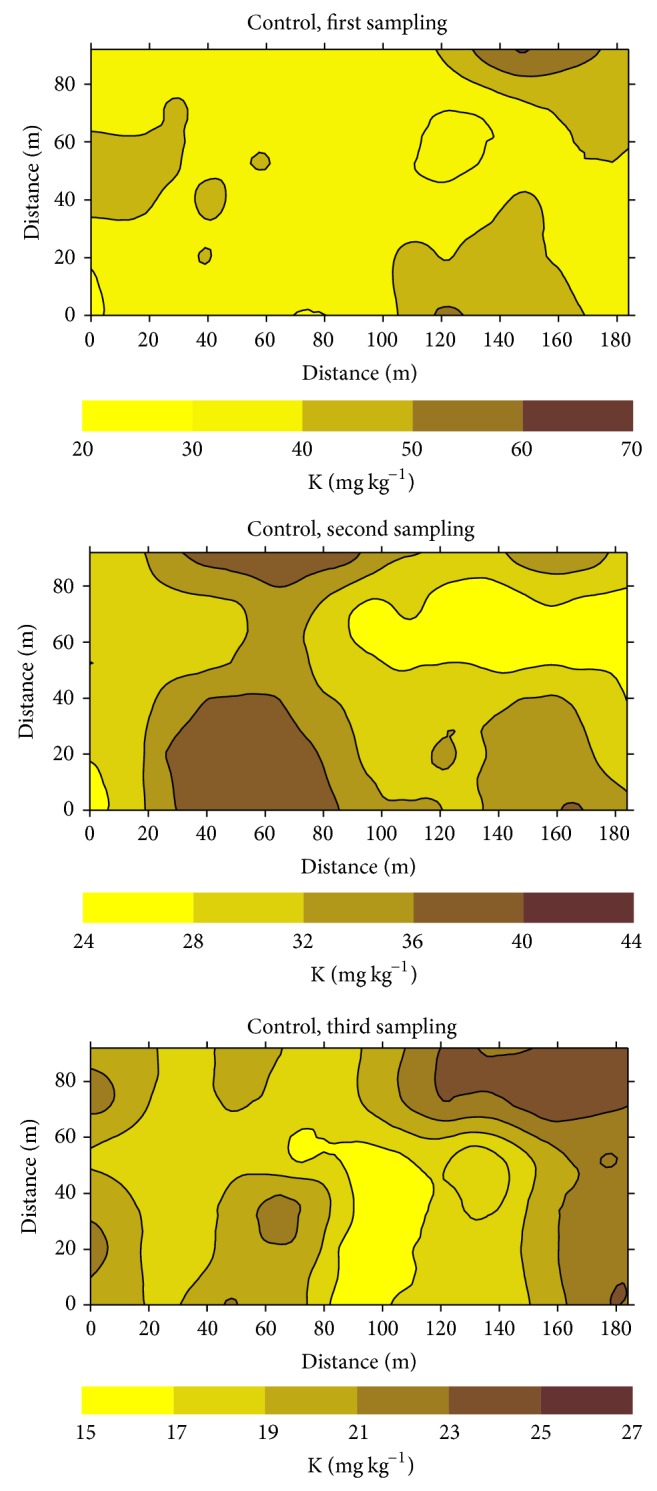
Kriging maps for Mehlich-1 extractable potassium (K) at the control treatment, for successive sampling dates.

Examples of kriging maps for ammonium-N, Olsen P, and Mehlich-1 K are shown in Figures 4, 5, and 6, respectively. The selected examples correspond to the three successive sampling dates of the control treatment. These maps illustrate areas with varied spatial and temporal concentrations of nutrient throughout the experimental fields. Concentrations of ammonium-N ranged from 11 to 29 mg kg^−1^, 17 to 38 mg kg^−1^, and 30 to 48 mg kg^−1^ at the first, second, and third sampling dates, respectively. Similarly Olsen P ranged from 3 to 9 mg kg^−1^, from 6 to 20 mg kg^−1^, and from 5 to 12 mg kg^−1^ in these sampling periods, whereas the respective ranges of variation for Mehlich-1 K were from 20 to 70 mg kg^−1^, 24 to 44 mg kg^−1^, and 15 to 17 mg kg^−1^. Differences between patches with the lowest and the highest concentrations ranged approximately between 1.5 and 3.5. Kriging maps also clearly illustrated the presence of small scale variability for NH_4_^+^-N, Olsen P, and Mehlich-1 K within each liming treatment (data not shown) and during each of the three sampling dates.

The patterns of spatial distribution of the three nutrients presented in the maps of Figures 4, 5, and 6 are characterized by small zones, that is, discrete patches, with heterogeneous values of the studied variables. For a given area, differences in concentration among sampling dates are clearly shown, so that areas with relative high or low macronutrient concentrations within each field were not stable throughout the rice growth period. Thus, there was a lack of temporal stability for the macronutrients studied, similar to that previously described for pH and Eh [17] and for Fe, Mn, and Zn [12, 13].

Our results also showed that seasonality changed the patterns of distribution of ionic species with different degrees of mobility such as ammonium, phosphate, or potassium. Subsequently, seasonal variability in soil macronutrient concentration (N, P, K) was not only present but potentially of agronomic importance.

Our macronutrient maps were based on an intensive sampling on a 50 × 11.9 m grid. Farmers cannot afford this level of investment in sampling and often rely on data from satellites or aircraft or from tractor-borne equipment. However, and in spite of significant technological advances, nowadays a lack of decision criteria still remains for an efficient site specific management of fertilizer inputs on several crops, and this is the case for lowland rice. To overcome this deficit nutrient maps based on intensive soil sampling are required. Therefore, kriging maps are useful for optimizing soil sampling and for delineating management units. They have been widely used in rainfed agriculture as a straightforward approach for precise management of phosphorus and potassium, since these nutrients show comparatively stable spatial distribution patterns in aerobic conditions [2, 3, 6, 7].

In our lowland paddy soil, marked seasonal changes in mean concentrations and in the spatial distribution of ions with wide differences in mobility such as ammonium-N, phosphate, and potassium were detected. This information may be potentially very useful both for site specific management and for environmental purposes. In general, recommendations for fertilizer application to rice crops are based on correlation between soil test values of the target nutrient measured on samples collected before flooding. The above results suggest that the efficiency of site specific management practices in rice fields would increase if the observed patterns of seasonal changes in macronutrient concentration are taken into account. This is in agreement also with recent work [20].

On the other hand, analysis of soil spatial variability also provides valuable knowledge for environmental management. For example, if only mean values of macronutrients were taken into account, the low concentrations of ammonium-N and available P and K across the rice growth period were unlikely to have any adverse effects on the environment; however the presence of spots with nutrient concentrations higher than the average could be critical for ponding water and ground water quality.

## 4. Conclusions

Mean values of ammonium-N, Olsen P, and Mehlich-1 K sampled during three different dates along the rice growth period, and under three dolomite treatments, in an acid paddy soil, varied both seasonally and as a function of the liming rate. Increasing rates of dolomite amendment increased ammonium-N, Olsen P, and Mehlich-1 K, both before sowing (aerobic conditions) and 28 days after flooding (anaerobic conditions). Meanwhile, after 56 days of flooding effects of dolomite amendment on the concentration of the studied nutrients were dissimilar.

Ammonium-N accumulated in anaerobic conditions and was highest at the third sampling date, 56 days after flooding. Olsen P availability increased with increasing pH after flooding, but this effect was reversed for pH values higher than 6.5 measured at the flowering stage of the rice crop. Mehlich-1 K steadily decreased along the growth period of rice.

The spatial variability of NH_4_^+^-N, P, and K on rice fields with three different rates of dolomite amendment and at three different crop stages was best described mainly by spherical models and otherwise with exponential models. The nugget to sill ratio ranged from 0 to 43.7% for all the data sets studied, showing strong to moderate pattern of spatial dependence. The small to moderate nugget effects also indicate that the sampling grid used was proper to reflect the spatial dependence of the studied macronutrients.

Kriging maps clearly showed the presence of small scale variability for NH_4_^+^-N, Olsen P, and Mehlich-1 K within each liming treatment and during each of the three sampling dates. Also the position of patches with high and low concentrations of the studied macronutrients changed between successive sampling dates, providing evidence of the lack of temporal stability in the patterns of spatial distribution. Seasonality in the spatial distribution of macronutrients should be considered as an important factor for an efficient site specific management.

## Figures and Tables

**Table 1:** Mean ± standard deviation for pH and Eh at three sampling dates and on three different lime treatments. (Mean values followed by a diffferent lowercase letter in the column and different capital letter in the row are significantly different).

Dolomite	1st sampling (before sowing)	2nd sampling (day 28)	3rd sampling (day 56)
pH			
0 kg ha^−1^	4.2 ± 0.09^aA^	5.7 ± 0.28^bB^	6.6 ± 0.24^cC^
620 kg ha^−1^	4.3 ± 0.07^aA^	5.8 ± 0.30^bB^	6.7 ± 0.22^cC^
1250 kg ha^−1^	4.4 ± 0.07^aA^	5.9 ± 0.24^bB^	6.8 ± 0.12^cC^
Eh (mV)			
0 kg ha^−1^	554.4 ± 7.4^aA^	−16.4 ± 19.7^aB^	−186.2 ± 31.9^aC^
620 kg ha^−1^	539.7 ± 10.6^aA^	−25.8 ± 15.5^bB^	−189.5 ± 29.7^aC^
1250 kg ha^−1^	532.4 ± 10.6^aA^	−30.1 ± 13.5^bB^	−210.3 ± 31.1^bC^

**Table 2:** Summary statistics for NH_4_^+^-N, P, and K concentrations on different sampling dates and liming treatments (Var.: variance; CV: coefficient of variation).

Lime (Kg ha^−1^)	Code	Mean (mg kg^−1^)	Var.	CV	Minimum (mg kg^−1^)	Maximum (mg kg^−1^)	Skewness	Kurtosis
Ammonium nitrogen, NH_4_^+^-N								

First sampling								
0	NH_4_-10	20.07^a,A^	14.67	19.1	11.16	28.80	−0.047	−0.405
625	NH_4_-11	28.87^c,B^	17.09	14.3	20.42	39.72	0.159	−0.435
1250	NH_4_-12	27.30^b,A^	30.56	20.3	11.62	43.63	0.064	0.856

Second sampling								
0	NH_4_-20	24.73^a,B^	15.29	15.8	17.09	35.28	0.319	−0.530
625	NH_4_-21	27.36^b,A^	11.21	12.2	21.43	37.38	0.654	−0.036
1250	NH_4_-22	28.50^c,B^	12.56	12.4	19.79	36.94	0.099	−0.775

Third sampling								
0	NH_4_-30	37.74^a,C^	24.14	13.0	28.84	48.52	0.433	−0.820
625	NH_4_-31	43.92^b,C^	31.23	12.7	30.38	55.38	−0.462	−0.251
1250	NH_4_-32	38.84^c,C^	14.25	9.7	29.60	48.54	0.126	−0.033

Phosphorus, P								

First sampling								
0	P-10	5.52^a,A^	1.75	24.0	2.50	8.04	0.041	−0.730
625	P-11	6.72^b,A^	2.03	21.2	3.74	10.72	0.450	−0.346
1250	P-12	8.57^c,A^	2.60	18.8	5.04	12.33	−0.160	−0.274

Second sampling								
0	P-20	10.36^a,C^	4.75	21.0	6.61	18.45	1.069	1.562
625	P-21	13.12^b,C^	6.50	19.4	7.30	18.43	0.012	−0.670
1250	P-22	11.93^c,C^	3.39	15.4	8.72	17.34	0.502	0.037

Third sampling								
0	P-30	7.14^a,B^	3.39	25.8	3.50	14.26	0.894	1.633
625	P-31	8.67^b,B^	2.72	19.1	4.79	13.17	0.194	−0.319
1250	P-32	7.45^a,B^	3.02	23.3	4.05	10.69	0.111	−0.883

Potassium, K								

First sampling								
0	K-10	38.15^a,C^	47.95	18.2	26.56	64.64	1.012	1.551
625	K-11	44.41^b,C^	36.93	13.7	34.26	65.21	0.822	0.897
1250	K-12	50.41^c,C^	35.78	11.9	38.24	70.53	0.380	0.387

Second sampling								
0	K-20	32.09^a,B^	23.54	15.1	22.01	42.35	0.004	−0.969
625	K-21	36.46^b,B^	86.97	25.6	19.86	59.87	0.277	−0.860
1250	K-22	40.44^c,B^	57.47	18.8	28.73	59.68	0.355	−0.649

Third sampling								
0	K-30	19.41^b,A^	7.48	14.1	12.43	25.30	0.019	−0.647
625	K-31	18.01^a,A^	5.78	13.4	12.42	23.95	0.137	−0.022
1250	K-32	17.25^a,A^	9.56	17.9	11.34	23.23	0.177	−1.038

Mean values followed by different small letters and different capital letters are significantly different (*P* < 0.05) for liming treatment and sampling date, respectively.

**Table 3:** Semivariogram model type, parameters for best fitted model (*C*_0_: nugget; *C*_1_: structural variance; *a*: range), and cross-validation indicators (*r* ^2^: determination coefficient; ME: mean error, NMSE: no dimensional mean square error) of the studied NH_4_-N, P, and K data sets.

Lime	Code	Model	*C*_0_	*C*_1_	*C*_0_/(*C*_0_ + *C*_1_)	*a* (m)	*r* ^2^	ME	NMSE
Ammonium nitrogen, NH_4_^+^-N									

First sampling									
0	NH_4_-10	Spherical	1.09	14.20	7.1	47.0	0.923	0.003	1.004
625	NH_4_-11	Spherical	6.37	10.41	38.0	45.1	0.663	−0.011	0.994
1250	NH_4_-12	Spherical	13.58	19.05	41.6	68.9	0.932	−0.016	0.926

Second sampling									
0	NH_4_-20	Spherical	0.00	15.57	0.0	40.9	0.876	−0.003	1.147
625	NH_4_-21	Exponential	0.00	12.25	0.0	59.5	0.912	−0.005	0.992
1250	NH_4_-22	Exponential	0.00	13.08	0.0	49.8	0.869	−0.019	1.000

Third sampling									
0	NH_4_-30	Spherical	1.95	24.15	7.5	53.2	0.639	−0.001	0.813
625	NH_4_-31	Spherical	0.00	33.96	0.0	71.9	0.907	0.002	0.922
1250	NH_4_-32	Spherical	2.60	13.92	15.7	84.6	0.972	−0.008	0.937

Phosphorus, P									

First sampling									
0	P-10	Spherical	0.23	1.59	12.6	51.7	0.835	−0.052	1.060
625	P-11	Spherical	0.61	1.54	28.4	64.0	0.836	−0.003	1.020
1250	P-12	Spherical	0.44	2.29	16.1	53.0	0.850	0.002	0.903

Second sampling									
0	P-20	Spherical	0.42	4.86	8.0	65.2	0.951	−0.000	1.097
625	P-21	Spherical	0.43	6.24	6.4	47.7	0.944	−0.019	0.996
1250	P-22	Spherical	1.53	1.97	43.7	76.3	0.908	−0.014	1.029

Third sampling									
0	P-30	Spherical	1.41	2.30	38.0	73.9	0.976	0.002	0.848
625	P-31	Spherical	0.74	2.11	26.0	57.7	0.843	0.027	0.855
1250	P-32	Spherical	0.89	2.25	28.3	60.0	0.912	0.001	0.941

Potassium, K									

First sampling									
0	K-10	Spherical	4.97	46.74	9.6	59.7	0.861	0.011	1.184
625	K-11	Spherical	6.55	33.12	16.5	54.2	0.903	−0.005	0.948
1250	K-12	Spherical	15.96	21.35	42.8	51.4	0.925	0.013	0.901

Second sampling									
0	K-20	Spherical	6.73	18.65	26.5	65.5	0.891	0.006	0.954
625	K-21	Spherical	14.61	75.20	16.3	52.3	0.887	0.049	0.863
1250	K-22	Spherical	16.56	43.79	27.4	58.9	0.783	0.004	0.782

Third sampling									
0	K-30	Spherical	1.46	6.66	18.0	63.1	0.899	−0.024	0.882
625	K-31	Spherical	2.46	3.71	39.9	68.2	0.921	0.003	1.075
1250	K-32	Spherical	3.52	6.84	34.0	76.8	0.960	−0.008	0.690
